# Quality of Palliative and End-Of-Life Care in Hong Kong: Perspectives of Healthcare Providers

**DOI:** 10.3390/ijerph17145130

**Published:** 2020-07-16

**Authors:** Eliza Lai-Yi Wong, Nicole Kiang, Roger Yat-Nork Chung, Janice Lau, Patsy Yuen-Kwan Chau, Samuel Yeung-Shan Wong, Jean Woo, Emily Ying-Yang Chan, Eng-Kiong Yeoh

**Affiliations:** 1 The Jockey Club School of Public Health and Primary Care, The Chinese University of Hong Kong, Hong Kong, China; nicolekiang@hotmail.com (N.K.); rychung@cuhk.edu.hk (R.Y.-N.C.); janicelau@cuhk.edu.hk (J.L.); patsychau@cuhk.edu.hk (P.Y.-K.C.); yeungshanwong@cuhk.edu.hk (S.Y.-S.W.); emilychan@cuhk.edu.hk (E.Y.-Y.C.); yeoh_ek@cuhk.edu.hk (E.-K.Y.); 2 CUHK Jockey Club Institute of Ageing, The Chinese University of Hong Kong, Hong Kong, China; jeanwoowong@cuhk.edu.hk

**Keywords:** palliative care, end-of-life, health service, healthcare providers, qualitative

## Abstract

Background: In response to population aging, there is a need for health systems to focus on care for chronic disease, specifically palliative care, while focusing on people-centered care. The objective of this study is to explore the healthcare system enablers and barriers to the provision of quality palliative and end-of-life care from the perspective of healthcare professionals. Materials and Methods: Using purposive sampling, fifteen focus group interviews and nine individual interviews involving 72 healthcare providers were conducted. Primary qualitative data were collected between May 2016 and July 2017. All recorded discussions were transcribed verbatim and analyzed. A thematic framework was developed. Results: The provision of quality palliative and end-of-life care is influenced by the interaction and integration of nine sub-themes under four identified themes: (1) political context; (2) organization setting; (3) support to patients, caregivers, and family members, and (4) healthcare workers and the public. Conclusions: Integration of palliative and end-of-life care is an important pillar of healthcare service to improve quality of life by addressing patients’ values, wishes and preference, and assist their family to handle challenges at the end stage of life. Further improvements to the service framework would be required, specifically in the political framework, multidisciplinary approach, and readiness and competence in healthcare workers and community. These were highlighted in our study as key components in service provision to ensure that patients can receive continuous and integrated care between hospitals and the community as well as dignified care at the end stage of life.

## 1. Background

Globally, the estimated number of adults in need of palliative care at the end-of-life (EOL) is over 19 million [1], and, in England and Wales, it is projected that in 2040 56% of people aged 85 and older will require palliative care [2]. Over the years, health systems around the world have increased palliative care services by establishing palliative care units and piloting various inpatient and community palliative care programs to increase the availability and access to the population [3]. Despite this, continued efforts would still be required to ensure that the quality and accessibility of palliative care services [2,4].

In Hong Kong, palliative care services under public hospitals were originally only offered to patients with cancer; however, following the provision of palliative care to patients with end-stage renal failure at Caritas Medical Centre in 2008, palliative care services are also available to patients with organ failure. In 2013, palliative care services covered 68% and 44% of cancer and patients with renal failure, respectively, with 90% of deaths occurring in public hospitals [5]. In order to reduce unnecessary hospital admissions and to encourage aging well in the community, several public hospitals collaborated with elderly homes to provide palliative care services to elderly residents, especially for those who are frail and/or suffering from dementia. A geriatrician-led community program known as “Enhanced community geriatric assessment team support to EOL patients in residential care homes for the elderly” (EOL-RCHE) has been piloted in four hospital clusters since 2015 [6]. Another local initiative “End-of-Life Care in Residential Care Homes for the Elderly” was piloted by the Hong Kong Association of Gerontology in 36 sub-vented care homes to support institutional older adults [7].

Internationally, a number of studies in Europe, United Kingdom, and United States attempted to explore the issues and challenges to palliative care provision. Three major themes were identified in term of political context, organizational deficiency, and implementation experience including readiness of individuals [8,9,10]. However, these findings are limited in Western culture, variations in challenges may occur according to local contexts due to different designs in healthcare systems and culture value. Hong Kong is a unique jurisdiction as it is a special administrative region of China and legacy of British Colony; thus, there is a need to examine the healthcare system in Hong Kong in order to enable the provision of integrated care to all patients. Findings could provide insight and additional body of knowledge for other jurisdictions in the development of palliative and EOL care. This study aims to explore the enabling and inhibiting factors in the implementation of palliative and EOL care for the elderly in Hong Kong from the perspectives of healthcare professionals.

## 2. Materials and Methods

### 2.1. Study Design and Participants

A qualitative study design was used to explore the in-depth experience of different stakeholders involved in providing palliative and EOL care in Hong Kong. In order to ensure representativeness of the study, practitioners, nurses, and various allied health professionals, who are involved in the implementation of palliative and EOL care for at least one year with different ranks as a proxy of seniority and clinical exposure from three hospital clusters, were purposively sampled from the potential list provided by the Hospital Authority. Eligible participants were invited through telephone and email to participate in the study. Participants who accepted the study invitation were able to choose the method of participation (focus group, face-to-face interview or telephone interview) most convenient to them for sharing experience and views.

### 2.2. Data Collection and Analyses

Focus groups discussions and interviews were conducted using a semi-structured interview guide at participants’ respective hospitals, and each focus group discussion and interview lasted 30–90 min. The interview guide was developed based on theoretical framework in literature findings and consisted of three themes: (1) political context, (2) organization setting, (3) healthcare workers and public [8,9,10]. All discussion sessions were conducted in Cantonese and guided by an interview guide, comprised of open-ended questions and probes to explore issues regarding the enabling and inhibiting factors in the Hong Kong healthcare system for service delivery and recommendations for future development. Prior to the discussion, the objectives of the research and discussion session were explained by the moderator, and participants’ demographic characteristics and written consent for audio recording were obtained. In addition to an experienced qualitative researcher acting as the moderator, other members of the research team were also present, acting as note-takers. The number of discussion sessions was guided by data saturation.

All recorded discussions were transcribed verbatim and verified by at least two members of the research team. Notes taken during each discussion session were also compared and verified by members of the research team. The transcripts of two focus group discussions were chosen at random to be analyzed independently by two researchers. Using the Grounded theory, emerging themes identified were reviewed and discussed, and a thematic framework was developed. Following the indexing of all transcripts into themes and sub-themes, the thematic framework was reviewed, where themes were split, combined, and/or discarded. All data was closely reviewed using the revised framework in case of any missed data. Themes and sub-themes were analyzed and compared for similarities and differences, and relationships between themes were made. All data collected were analyzed using NVivo10 (QSR International, Burlington, MA, USA).

## 3. Results

**Figure 1:**
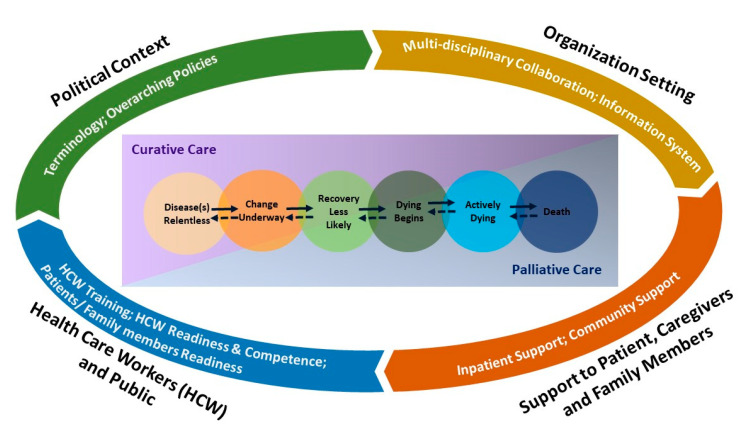
Thematic framework of palliative and end-of-life care service model.

A total of 15 focus group interviews and nine individual interviews were conducted between May 2016 and July 2017 among 72 healthcare professionals, including 14 (19.4%) practitioners, 16 (22.2%) nurses, and 42 (58.3%) allied health professionals. The demographics of healthcare professionals are shown in Table 1. While the stages from disease diagnosis to death may be progressive or regressive, the provision of quality palliative and EOL care is influenced by the interaction and integration of nine sub-themes under four identified themes: (1) political context; (2) organization setting; (3) support to patients, caregivers, and family members; and (4) healthcare workers and public (Table 2). The thematic framework developed based on the findings from this study is shown in Figure 1.

### 3.1. Political Context

#### 3.1.1. Terminology on Palliative and End-Of-Life Care

Palliative and end-of-life care is well in place for patients with cancer and kidney failure, which could be used as a service model to expand the elderly; however, the definition of care delivery is not well aligned in different disciplines. Due to the presence of various palliative and EOL services provided by various stakeholders, there is often confusion in the terminology. As palliative care services were generally only offered to cancer patients and those with end-stage organ failure, and the current EOL-RCHE program (End-of-Life–Residential Care Homes for the Elderly) mainly targets frail elderly, there is a general misconception that palliative care refers to care for cancer patients and EOL care refers to patients with other life-limiting conditions. However, one participant expressed that these elements “are essentially the same” and that “the terminology can be confusing”. Furthermore, the timeframe that the term “EOL” refers to may also vary between contexts. EOL may refer to any time between the last year of life and the dying moment, where the level of care may differ significantly.

*“There is actually a slight difference between “EOL care” in the UK and “EOL care” in other countries. Some, refer to patients’ last few days and others may be talking about last few months, or even year. So we don’t actually have standardized terminology.”*

*[I5, doctor]*

There may be concerns among patients and family members that the initiation of EOL care services may suggest that the patient is at the end stage of life and that death is approaching. Moreover, as there is an option within EOL care services to withdraw life-sustaining treatments, there may be misunderstandings on the objectives of EOL care.

*“We heard in some discussions that there are misconceptions among citizens that EOL refers to giving up treatment…”*

*[I FGD5, dietitian]*

#### 3.1.2. Overarching Policies for Palliative and End-Of-Life Care

In order to provide person-centered care and enable patients to be cared for with dignity, efforts should be made to facilitate aging and dying in place for patients who would like to be cared for in the community. However, there is a lack of capacity in the healthcare system to meet such needs.

*“… the system isn’t ready… regarding dying at home, the system is not well coordinated. This includes not having good service to support [dying at home].”*

*[I7, doctor]*

Furthermore, overarching guidelines and policies for the provision of palliative and EOL care are inadequate. Piecemeal palliative and EOL care programs are established by different organizations, with different service guidelines and frameworks. This leads to fragmentation of services; some patients may receive overlapping services, while others may not receive any service.

*“…the government does not have clear-cut policies or directions, actually on elderly care as a whole… The government wants to focus on elderly health services, EOL care services, those sorts of things. But in my opinion, they are very [disease-specific], firstly. Secondly it is very fragmented.”*

*[A FGD1, physiotherapist]*

### 3.2. Organizational Setting

#### 3.2.1. Multidisciplinary Collaboration

In order to ensure that the needs of all patients are met, multidisciplinary teams of physicians, nurses, and allied health professionals are involved in providing holistic care to patients. One of the participants indicated that case conferences take place every week, where “a multidisciplinary team of physicians, nurses, and allied health professionals would gather together… and review the cases”.

However, comments from participants may suggest that the provision of holistic care by multidisciplinary teams may be limited to hospital settings. Community support, specifically for patients living at home, is mainly provided by home care nurses, with limited support from physicians and allied health professionals, and only focuses on healthcare.

*“Currently, under palliative care services, home care support is mainly [provided by] home care nurses, with the support from physicians, mainly home care nurses. But I feel that more can be done in the social [care] aspect, because we feel that there is a gap… But it is still inadequate, because their manpower is limited, so they could only focus on their health.”*

*[B FGD2, medical social worker]*

*“Of course having a team is very important because sometimes one person may not be able to see things in all aspects... If [the patient] is under a multidisciplinary team or at least able to receive advice from a second discipline, the interaction may be able to provide a better service.”*

*[I8, doctor]*

*Similarly, although preparatory measures were carried out to enable elderly home staff to provide care in elderly homes, coordination between hospital and elderly home staff may be limited. Inadequate collaboration between medical and social sectors hinders the provision of integrated care, as one of the participants expressed fragmentation of services provided by different sectors and organizations.*

*“…right now there are loads of services, Social Welfare Department is working on [aging], Department of Health is working on it, HA [Hospital Authority] is working on it, non-governmental organizations are working on it, it is actually very fragmented, and everyone is doing their own thing…”*

*[C FGD1, physiotherapist]*

#### 3.2.2. Information System

The sharing of medical records within hospitals through a computer system facilitates the provision of a continuum of care. Through the information system, staff in other medical departments, specifically in Accident and Emergency (A&E), can recognize patients under palliative and EOL care who may have expressed their wishes regarding medical treatments through Advance Directive (AD), Advance Care Planning (ACP), and/or Do-Not-Attempt Cardiopulmonary Resuscitation (DNACPR), and therefore reduce unnecessary interventions.

*“it [AD/ACP/DNACPR documents] is in the CMS [Clinical Management System], so it would be able to alert [A&E staff]. If an A&E staff has assessed the case and believe that the patient does not need to be admitted to [acute hospital] ward, then (s) he would know, and would send the patient to [convalescent hospital] ward as soon as possible.”*

*[C FGD10, nurse]*

Participants have, however, pointed out flaws in the information system, revealing that some information may only be accessed by certain departments, staff, and hospitals. Furthermore, as certain information including the activities carried out by allied health professionals and social care providers are not recorded in the system, healthcare providers may not be fully aware of the types of care received by the elderly.

*“…so I think in the CMS platform, our allied health notes would not be seen because we hand write all of them. Once it is written, it is then stored in the cabinet, so physicians do not actually know about our progress…”*

*[A FGD3, physiotherapist]*

### 3.3. Support to Patients, Caregivers, and Family Members

#### 3.3.1. Inpatient Support

Due to high demands of patients requiring palliative care, inadequate resources, especially in the health workforce, may hinder the capacity to provide appropriate care to all patients. One of the participants expressed that initiation of palliative care may sometimes be delayed due to lack of resources.

*“Sometimes at the acute ward, [health providers] may feel that [the provision of palliative care] is not their priority… they are already very busy doing rescue work.”*

*[I3, doctor]*

*“…even in our oncology center, there may be patients who would start palliative care at a very late stage… this is actually not ideal. It should be started sooner, the sooner the better. ‘The sooner the better’ is linked with resources.”*

*[I8, doctor]*

*Consultative teams of palliative care specialists are established to provide support to healthcare providers at acute settings. However, participants have indicated that further improvements could be made, including increasing the manpower of different disciplines in the consultative teams.*

*“Different hospitals have consultative teams, but right now it is not very comprehensive. The staff manpower of the teams are insufficient…not all hospitals have a consultative team to provide services, and even if they do, provision would not be comprehensive. It may require nurses, physicians, and allied health professionals to support, to consult patients and to give advice.”*

*[I7, doctor]*

#### 3.3.2. Community Support

Though there is a communication platform between the hospital team and community team, similar to the hospital setting, there is also inadequate support for patients in the community. The lack of medical expertise and equipment to care for patients living at home may result in pain and discomfort for patients, as well as stress and burden to caregivers and family members. One of the participants noted that the lack of community support may lead to high demands for inpatient palliative care.

*“…the problem we are facing right now is that the support outside of hospitals is inadequate... Actually, there are some patients [who], you can say, medically, can be discharged... Technically they do not require hospital-level care, but most of the time; the tendency is that their family [size]… may be very small, only the couple. Also, the partner most probably won’t be young, and (s) he would not have any experience of medical care and would find it difficult to look after the patient. Therefore the patient is staying in the hospital.”*

*[I5, doctor]*

One of the participants expressed that increasing support to patients living at home may be able to improve patients’ dignity and quality of life.

*“…if we are able to support [patients at home], their quality of life may be able to improve. For example, if they are able to be more functional in their lives, then they may also gain dignity.”*

*[D FGD3, occupational therapist]*

The inability to care for patients in the community would mean that patients would have to go to the hospitals for consultations and further interventions. However, there is limited transportation for patients who are wheelchair- or bed-bound.

*“…there basically isn’t any transportation. Because you can’t, per usual, take the bus… They may be wheelchair-bound [and need] special transportation, but right now… it’s very difficult to find one.”*

*[A FGD6, occupational therapist]*

*“Because the only service that does not require extra charge is NEATS [Non-emergency Ambulance Transfer Service]. But the service of NEATS is limited… They may only transfer four, five [patients at a time]… there are also criteria. You have to either be living alone or [live in places not accessible by elevators]… or you require long-term oxygen, only then would they pick you up. Therefore if a typical elderly does not fall into [these categories] then (s) he would have to find his/her own transportation.”*

*[B FGD6, physiotherapist]*

### 3.4. Healthcare Workers and Public

#### 3.4.1. Staff Training and Skill Transfer

In order to ensure that palliative care are provided to all patients, palliative care training and skills transfer are provided by palliative care specialists to non-specialists involved in the provision of care under the EOL-RCHE program.

*“Actually the current situation [of the EOL-RCHE program] is that palliative care staff would, to a certain extent, enable geriatric staff to manage the needs of patients. We are talking about some sort of teaching, skill transfer and even sending a member of staff over to do some coaching…”*

*[I5, doctor]*

However, some participants, specifically nurses under the program revealed that training was not adequate and expressed their wishes to receive more training and skill transfer, specifically on counseling skills and the provision of psychosocial support.

*“The staff training prior to the program is actually very limited, but as soon as the program starts, [they] already have to start doing intensive work, talking about life and death. It actually isn’t easy, having to do it based on a couple of training. You really need some sort of talent to be able to do that.”*

*[D FGD12, nurse—APN]*

#### 3.4.2. Readiness and Competence of Healthcare Staff

Culture of providing palliative and EOL care in elderly care provision is developing with the government’s overarching policy: ageing and dying well in place. However, due to the lack of palliative care training for non-palliative care specialists, healthcare providers involved in the provision of EOL care may not be ready to experience death and dying and provide comprehensive care to patients and their family members.

*“…I believe it’s their years of experience, and also their views on care at the EOL or on death. Because…they may have had bad experience regarding death. This would actually affect their views regarding care at the EOL. Let’s not talk about having to talk to others, they can’t even overcome themselves, how can they communicate with the family members [on issues regarding death].”*

*[A FD10, nurse]*

Most elderly homes are still unable to facilitate patients who may wish to spend their last moments and die in elderly homes.

*“Most of the time elderly home staff may not have the knowledge or skill to monitor the elderly, or they may—as soon as they feel that something isn’t right—they would [immediately] send the patient to A&E.”*

*[E FGD 3, social worker]*

Therefore, training of community care staff is also essential to ensure the provision of quality EOL care in the community.

*“We can actually do a bit more in our role, which is to train elderly home staff or community center staff…if staff have more understanding regarding EOL care, then they would not be so alarmed. If there is regular training, then they may be able to help calm the patient, not to be so anxious, worried or depressed.”*

*[C FGD4, clinical psychologist]*

#### 3.4.3. Readiness of Patients and Family Members

Readiness of patients and family members may bring an impact the provision of care for the dying. Due to limited land for cemeteries in Hong Kong, government advocates green burial as a more environmentally friendly and sustainable means of handling human ashes, which raises public awareness on the arrangement of cremated ashes as well as EOL care in family discussion. However, participants revealed that the taboo around death may hinder conversations among healthcare providers, patients, and family members regarding future care plans and other EOL arrangements.

*“Ultimately, Chinese individuals are very concerned with the dying taboo, it is rarely mentioned. I noticed in my clinic, some patients may not necessarily be ready to discuss about it.”*

*[I7, doctor]*

*“I noticed that most of the time, patients are not unwilling to talk [about EOL issues], but most of the time it is the family members, or specific family members who may not be willing to talk. They would stop others from talking. These are actually very specific cases, but as long as these sorts of people are present, family discussions become complicated.”*

*[I5, doctor]*

Most of the participants agreed that open culture and public education on life and death preparedness is necessary and important to facilitate the delivery of palliative and EOL care. Positive culture and education would enable better understanding of death, break the impasse in family, and not view it as a taboo.

*“they [family members] have actually voiced out that they don’t know how to handle. They do not have the knowledge to handle. Let’s say grasping, they believe that the patient is suffering, would [sending the patient to the hospital and] giving the patient oxygen relive their suffering or should the patient stay at home? So they would hesitate, unable to make the decision... If the patient can pass away comfortably, in their sleep, then that’s great. But if the patient is in pain and suffering, actually family members would not know how to handle it…”*

*[E FGD6, physiotherapist]*

*“But the situation right now, I think, is that the whole culture needs to be educated, that death is nothing to be scared of… death is just a transition. Many of this is really education, for example now that we have experienced the death of family members, it’s actually nothing to be afraid of.”*

*[I FGD5, dietitian]*

*“I agree with what we mentioned about earlier; education, to enable the community to gain knowledge on the topic, also to prevent unnecessary depression.”*

*[B FGD4, medical social worker]*

## 4. Discussion

### 4.1. Palliative and End-Of-Life Care Service Framework

Findings from this study indicate a need for improvements in the overarching service framework in healthcare system for the provision of care to the elderly. First, relevant terminology should be clearly defined and standardized among all stakeholders. Confusion in palliative and EOL care terminology has also previously been identified. In the independent review of United Kingdom’s (UK) Liverpool Care Pathway, Neuberger et al. (2013) [11] criticized the lack of clarity over the term “EOL”, as it could cover a broad period of time, from the last year of life to the dying moment. Moreover, Lo et al. (2010) [12] reported that there is a general lack of familiarity among healthcare professionals on the term and concept of palliative care. Therefore, terminology should be standardized and used in the service framework to ensure that appropriate services are provided. Second, guidelines and policies should be developed to facilitate the provision of integrated and coordinated care. Fragmentation of services and poor communication between services providers have been identified in our study and in international studies as issues related to the provision of integrated care [13,14,15,16]. Integration of services should focus on both health and social needs, using an interdisciplinary approach with teams of physicians, nurses, allied health professionals, and other social care providers. Care for patients in all settings—in hospitals and in the community—should also be considered. Furthermore, an information system should be developed to facilitate the communication and coordination between all stakeholders and to ensure that a continuum of care is provided, especially following the transition from hospital to community settings, and vice versa.

### 4.2. Readiness and Competency on Palliative Care and End-Of-Life

The general lack of knowledge among the public on issues related to palliative care and EOL identified in this study may be due to the cultural taboo of death and the view of palliative care as giving up [17,18]. A previous study revealed physicians’ perception that patients will react negatively and lose hope as a barrier to the initiation of palliative care [19]. This signifies the importance of public education in ensuring that appropriate care is provided to patients, according to the patient’s wish, by encouraging conversations among patients, family members, and healthcare providers. Family is an important context to open up the discussion of EOL care about preference and expectation. Taboo of death is one of key factors in Chinese culture that hinders the discussion; our study also reveals that family members are more reluctant than the patients in the discussion due to inadequate knowledge and communication skill. They want to avoid decision-making in the crisis situation due to possible feelings of guilt [20]. On the other hand, patients may choose to defer decision-making to family or healthcare professional due to limited capacity of decision-making; therefore, it is important to consider the family dynamic and not only plan around the patient [21]. In United Kingdom, collaborative approach between doctor and patient with the support of family is introduced to open and facilitate clear communication about illness and death and discussion about preference in care pathway [22]. Balance between individual preference and family dynamics is likely different in different families, levels of health literacy, religious beliefs, cultures, and stage of disease journey. It is important to plan the care according to these factors.

Similarly, healthcare providers, specifically non-palliative care specialists in hospital, elderly home, and community settings, may also have inadequate knowledge regarding the provision of palliative care. This is could be due to lack of palliative care training in health and social care professional education. In general, healthcare professionals may not have experience in EOL care, causing delay in referrals and moral stress in the discussion. This is consistent with other findings which also reported that despite having medical knowledge, healthcare providers may lack the skills and knowledge to provide palliative care services [8,13,15,23,24]. It echoes with the findings of a study in Hong Kong about the association between staff training in nursing homes and their willingness, competence, and self-care [7]. Findings from a previous study revealed that higher levels of palliative care knowledge in nursing homes are associated with better quality of care [25], education to care providers should be enhanced. Education focusing on both practical and emotional elements can facilitate care providers to face and provide care to the dying, as well as the bereaved [26]. Consultations and moral support from palliative care specialists would also facilitate the accessibility of service provision [8,12,27]. Demand on palliative and EOL care will dramatically increase with the aging population, and mandatory training on both communication skills and service delivery is strongly recommended.

### 4.3. Context for Enabling Aging and Dying in Place

Compared to other countries, such as the Unites States and the Netherlands, the percentage of deaths outside of hospitals in Hong Kong is very low [28]. The findings from this study indicated that family members and elderly home staff may lack adequate knowledge and capability to support the dying patient in the community. This is consistent with international findings which revealed that further support from medical experts would be required to enable patients to be cared for and spend their last moments of life in the community [29,30,31]. In addition, family members and elderly home staff may not be emotionally ready to provide care to the dying, further emphasizing the importance of training and public education [32].

Tailored to local context, findings from this study revealed that transportation between hospitals and the community may be a barrier to aging and dying in place in Hong Kong. This could be due to inadequate transportation for non-emergency care and legal requirements for ambulance staff to perform resuscitation [6]. The lack of appropriate transportation may result in lack of integration between hospitals and the community. Therefore, in order to facilitate elderly patients to stay in the community for as long as possible and, when needed, be transported to institutions for medical care without unnecessary interventions, issues related to the transition between hospital and the community would need to be resolved.

## 5. Limitations

The healthcare system is complex and dynamic, and service provision among different stakeholders, perspectives of social care providers, and patients should all be considered. This study focusing on the experiences from healthcare providers may limit the overview of the healthcare system enablers and barriers on the implementation of palliative and EOL care. Thus, our next step would be to explore the views from professionals working in social sectors and patients and their family members. Furthermore, due to local culture, healthcare financing, and the practical context of service delivery, the findings and implications from this study may not be fully applicable to other countries.

## 6. Conclusions

In response to the aging population, there is a need for health system to expand their focus by embracing palliative care and work towards integrated person-centered care. These study findings highlight key community and healthcare system factors that should be considered by policy, program planners and implementers in the design of palliative and EOL care services by identifying some of the facilitators and challenges in responding to the needs of the diverse elderly in Hong Kong. First, overarching framework and policies should also be developed with clearly defined and standardized terminology to be applied in all settings. Furthermore, enhancement of a multidisciplinary approach and information sharing in organizational setting is important to link between institution and community. Last, care provider training, competence acquisition, and cultural change through public education should be further developed to enable life and death conversations and ensure that patients’ expressed wishes are fulfilled. To align with the government’s actions to promote aging in place, ensuring that dying patients are able to stay in a familiar environment for as long as possible while receiving comfort and dignified care, further support for caregivers and family members should also be provided to ensure that needs are increasingly met for supporting care recipients at the end stage of life. Palliative and end-of-life care present many challenges for service providers, as well as for patients and their families as identified by further works from the perspective of socio-ecological which provide additional insight for the design of health system [33].

## Figures and Tables

**Table 1:** Demographic characteristics of participants.

Demographics	*n* (%) [*N* = 72]
Gender	
Male	32 (44.4)
Female	40 (55.6)
**Profession**	
Doctors	14 (19.4)
Nurses	16 (22.2)
Allied health professionals	42 (58.3)
*Clinical psychologist*	*3 (7.1)*
*Dietitian*	*4 (9.5)*
*Ear, nose, and throat*	*1 (2.4)*
*Medical social worker*	*6 (14.3)*
*Occupational therapist*	*8 (19.0)*
*Physiotherapist*	*13 (31.0)*
*Podiatrist*	*4 (9.5)*
*Prosthetic and orthotic*	*1 (2.4)*
*Radiologist*	*1 (2.4)*
*Speech therapist*	*1 (2.4)*
**Years of experience**	
<3	2 (2.8)
3–5	5 (6.9)
6–10	4 (0.6)
11–15	10 (14.9)
≥16	46 (63.9)
Refuse to answer	5 (6.9)
**Seven Hospital clusters**	
Hong Kong East Cluster	16 (22.2)
Hong Kong West Cluster	1 (1.4)
Kowloon East Cluster	18 (25)
Kowloon Central Cluster	2 (2.8)
Kowloon West Cluster	1 (1.4)
New Territories East Cluster	27 (37.5)
New Territories West Cluster	7 (9.7)

**Table 2:** Enablers and barriers in the implementation of palliative and end-of-life care.

Theme	Subtheme	Enablers	Barriers
Political Context	Overarching policies for palliative and end-of-life care	Patient-centered care and aging and dying in place is advocated	Lack of overarching guidelines and policies to align piecemeal programs
Organizational Setting	Multi-disciplinary collaboration	Multi-disciplinary approach is adopted in hospital	Community care is delivered only by nurses, with limited multidisciplinary support
	Information system	Computer system to share medical records with patient’s preference alert within and between hospitals is well in place	Flaws in information system in which certain information is accessed by different types of professionals and level of seniorities
Support to patients, caregivers, and family members	Inpatient support	Consultative teams of palliative care specialist is established to provide inpatient support	Delayed delivery due to limited manpower in consultative team
	Community support	Communication between hospital and community nurse for the delivery of care at home	Inadequate of medical expertise and equipment to care for patients at home Inadequate transportation facilities for patients in wheelchair or bed-bound
Healthcare workers and public	Staff training and skill transfer	Palliative care training and skill transfer provided by palliative care specialists to non-specialists	Inadequate training due to limited manpower in palliative care specialists
	Readiness of patients and family members	Increased public awareness and discussion of EOL in family with government’s advocacy of green burial	Taboo of death hinders conversations among patients, family members, and healthcare providers

## Data Availability

The datasets used and/or analyzed during the current study are available from the corresponding author on reasonable request.
